# Picophytoplankton size and biomass around equatorial eastern Indian Ocean

**DOI:** 10.1002/mbo3.629

**Published:** 2018-04-15

**Authors:** Yuqiu Wei, Jun Sun, Xiaodong Zhang, Jing Wang, Ke Huang

**Affiliations:** ^1^ Institute of Marine Science and Technology Shandong University Jinan China; ^2^ Research Centre for Indian Ocean Ecosystem Tianjin University of Science and Technology Tianjin China; ^3^ Tianjin Key Laboratory of Marine Resources and Chemistry Tianjin University of Science and Technology Tianjin China; ^4^ State Key Laboratory of Tropic Oceanography South China Sea Institute of Oceanology Chinese Academy of Sciences Guangzhou China

**Keywords:** biomass, cellular size, circulation and water mass, Indian Ocean, picophytoplankton

## Abstract

The cellular size and biomass of picophytoplankton were studied by flow cytometer during spring monsoon (March–May of 2015) in equatorial eastern Indian Ocean. We established an empirical relationship between forward scatter and cellular size to address the size and biomass of picophytoplankton. Results indicated that mean cell diameter of *Prochlorococcus* (0.60 μm) was the smallest, and then followed by *Synechococcus* (0.98 μm) and picoeukaryotic phytoplankton (1.05 μm). Thereafter, the biomass converted by abundance reached 0.64 μg·C·L^−1^ for *Prochlorococcus*, 0.34 μg·C·L^−1^ for *Synechococcus*, and 0.20 μg·C·L^−1^ for picoeukaryotic phytoplankton. Additionally, the distinct biomass contribution of picophytoplankton appeared to be affected by abundance, but not changes in cellular size. Vertically, the cellular sizes of picophytoplankton were remarkably small in upper waters, which was predominantly controlled by the nutrient availability. In contrast, they were larger in deeper waters, which was primarily attributed to the combined effects of low temperature and reduced light availability. Spatially, under the influence of high nutrient concentration induced by the different circulations and coastal upwelling, slightly high carbon biomass of picophytoplankton was observed around the coastal zones of Sri Lanka island and Sumatra, as well as the southern Bay of Bengal.

## 1 INTRODUCTION

Picophytoplankton (<2 μm) mostly composed of *Prochlorococcus*,* Synechococcus* and picoeukaryotic phytoplankton have essential roles in primary productivity in tropical and subtropical oligotrophic oceans (Stockner, 1988). Observations in the oligotrophic Pacific Ocean and Atlantic Ocean have shown that picophytoplankton accounts for approximately 60%–80% of the total primary productivity (Campbell, Liu, Nolla, & Vaulot, 1997). Since their considerably high biomass and contribution to marine primary production, picophytoplankton has been known to have large impacts on ocean ecosystem and biogeochemical cycles (Flombaum et al., 2013). The tropical Indian Ocean forms the major part of the largest warm pool on the earth, and its interaction with the monsoon plays an important role in shaping complex circulation systems on both regional and global scales (Wang, Xie, & Carton, 2004). Furthermore, the variability of nutrients, biomass, and primary production in the Indian Ocean induced by changes in physical forces have been investigated by a number of studies (McClanahan, Maina, Graham, & Jones, 2016; Roxy et al., 2016; Siswanto, 2015), which usually showed that the variability in phytoplankton standing stocks and primary production are closely related to the circulations and water masses. Although the Indian Ocean is considered as one of the largest oligotrophic areas, it has received far less attention than other oceans, particularly in terms of the size and biomass of picophytoplankton. Thus, presenting their size and biomass is critical to understand the contributions to carbon cycles of these special taxa in the Indian Ocean.

Thus far, flow cytometer (FCM) can help us to address the size and biomass of picophytoplankton at high frequency according to their cell morphological properties and fluorescence when the high‐sensitive protocol was used. For FCM, light scattering at different angles are related to the function of particle volume and secondarily shape (Latimer, 1982). However, an empirical calibration between cell diameter and side scatter (SSC) was performed to roughly estimate equivalent spherical diameter and cellular biovolume of picophytoplankton (Calvo‐Díaz & Morán, 2006; Chen et al., 2011). The range of picophytoplankton cell diameter, in general, used to establish the empirical relationship between cellular size and SSC is still critical (Gasol & Del Giorgio, 2000). Light scattering efficiency of picophytoplankton cell is a complex function of its size, structure, and refractive index, even different FCM and fixatives may yield significantly different scatter diagrams of the same sample as a function of relatively minor changes in detection geometry (Gasol & Del Giorgio, 2000). Consequently, Allman, Hann, Manchee, and Lloyd (1992) pointed out that cell diameter and light scattering should break down when comparing different species. According to the Mie theory, when particle diameter extends from 0.2 μm up to 2–3 μm or more, forward scatter (FSC) is the signal which is the most sensitive to cellular size, with a diameter *d* dependence of FSC in *d* ^4^‐*d* ^6^ (Morel, 1991). For example, the converting mean FSC to cell sizes for *Synechococcus* was done by fitting a power relationship with laboratory calibrations (FSC = *a* × Diameter^*b*^), and the exponent (*b*) was found to be 5.4, which was reasonably close to that determined by Mie light scattering theory (DuRand, Olson, & Chisholm, 2001); FSC versus biovolume data of *Synechococcus* reported a value of *d* ^5.1^ (Chisholm 1992); an FSC variation for *Prochlorococcus* during the daytime as a doubling in the average volume of the prokaryotes indicated a value of *d* ^5.4^ (Binder, Chisholm, Olson, Frankel, & Worden, 1996); the FSC related to particle sizes of reference beads for picoplankton led to the value around *d* ^5^ (Blanchot, André, Navarette, Neveux, & Radenac, 2001). Collectively, a strong correlation between FSC and cellular size has been determined by laboratory cultures of reasonably spherically shaped cells (DuRand et al., 2001; Olson, Zettler, & Anderson, 1989), despite small changes in refractive index (DuRand & Olson, 1998). Moreover, Koch, Robertson, and Button (1996) presented that FSC is chosen over SSC because of its far greater signal intensity to subcellular structure according to the theoretical basis of their approach. Actually, the relationship between FSC and picoplanktonic size has been carried out for several decades, even always involving bacterioplankton. Typically, Robertson and Button (1989) have made use of FSC to estimate bacterial size and proposed a good relationship between FSC and bacterial volume. However, some recently published papers often showed that the same large dispersion of beads and target cells in FSC limited the application of FSC and weakened its relationship with cellular size. To capture light scatter in forward angles and increase the sensitivity of this parameter, more instruments have been equipped with photomultiplier tubes (Gasol & Del Giorgio, 2000). This led Blanchot et al. (2001) to attempt a practical way for the estimation of mean cellular size what the relationship between the mean FSC and cellular diameter was determined by the power law empirically, which was assumed to stand for the mean FSC and diameters relative to those of the beads (*d*_cell_ = *d*_bead_(FSC)^1/5^), respectively.

In this study, we established an empirical relationship between FSC and cellular size to address the cellular size and biomass of picophytoplankton, and then to understand more clearly whether and how the contrasting environmental conditions affect their variations in equatorial eastern Indian Ocean. More specifically, will the expected environmental conditions induced by the complex circulations and water masses change the cellular size and biomass of picophytoplankton?

## 2 MATERIALS AND METHODS

### 2.1 Sampling strategy

**Figure 1:**
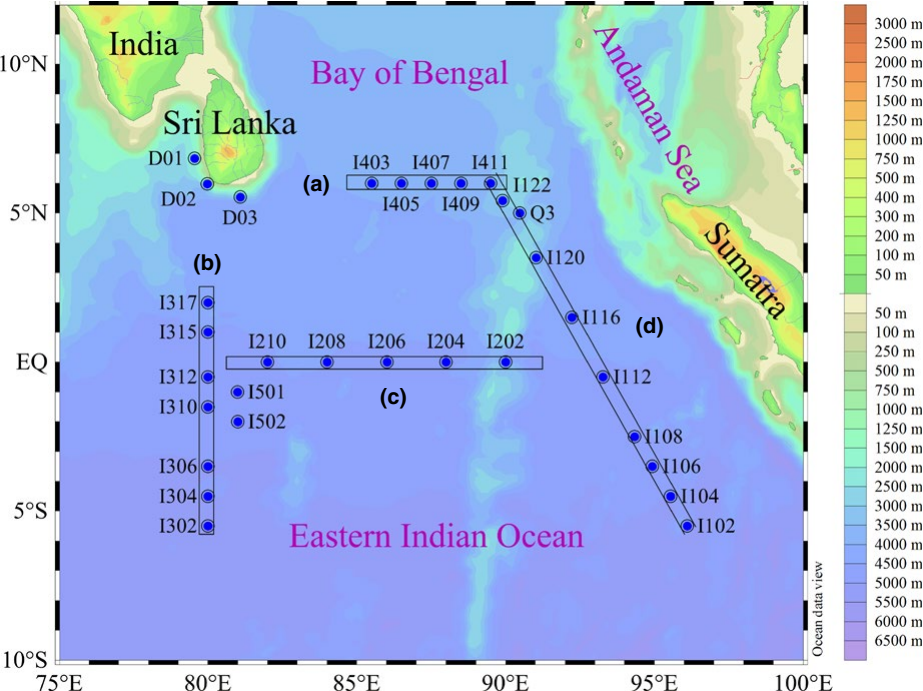
Study area and sampling stations. Four main transects (A–D) covered the entire eastern Indian Ocean were highlighted

This cruise was conducted on the R/V ***Shiyan I*** during spring 2015 (March 21–May 15) in equatorial eastern Indian Ocean (EIO; 6.8°N ~5.5°S, 79.5°E ~96.1°E) as shown in Figure 1. Our study area covered the entire equatorial EIO, and 31 stations were established. In addition, four selected transects were highlighted in this study. At each station, seawater samples were collected from seven depths within the upper 200‐m water column using 12‐L Niskin bottles equipped with a Sea‐Bird CTD (Conductivity, Temperature and Depth; SBE 19 Plus) rosette sampler. Photosynthetically active radiation (PAR) was measured by an RBR sensor (XRX‐620). The euphotic depth was defined as the depth of 1% surface light penetration. Temperature and salinity were recorded at the same time.

Seawater samples for picophytoplankton analysis by FCM were preserved on board with paraformaldehyde (1% final concentration). To avoid loss of resolution and changes in cell size due to fixation or freezing, FCM samples were kept in the dark without treatment at room temperature for 10–15 min, and then quickly freeze‐trapped in liquid nitrogen until analysis in the laboratory (Den Engh et al., 2017; Sommaruga, Hofer, Alonso‐Sáez, & Gasol, 2005).

Samples for nutrient analysis were filtered using 0.45‐μm cellulose acetate membrane filters, and then immediately refrigerated at −20°C for further analysis. Nutrient concentrations including ammonium, phosphate, nitrate, nitrite, and silicate were performed by a Technicon AA3 Auto‐Analyzer (Bran + Luebbe) according to the classical colorimetric methods. Dissolved inorganic nitrogen (DIN) defined as ammonium+nitrite+nitrate was analyzed using the copper‐cadmium column reduction method. Dissolved inorganic phosphorus (DIP) and silicate (DSI) were measured using the typical spectrophotometric methods (Dai et al., 2008; Guo et al., 2014). In all ranges of tested low nutrient standards, the AA3 was more precise and more accurate and showed lower detection limit for all channels: 0.018 μmol·L^−1^ for DIP, 0.009 μmol·L^−1^ for nitrate + nitrite, and 0.012 μmol·L^−1^ for DSI (Dafner, 2015).

### 2.2 Flow cytometry analysis

**Figure 2:**
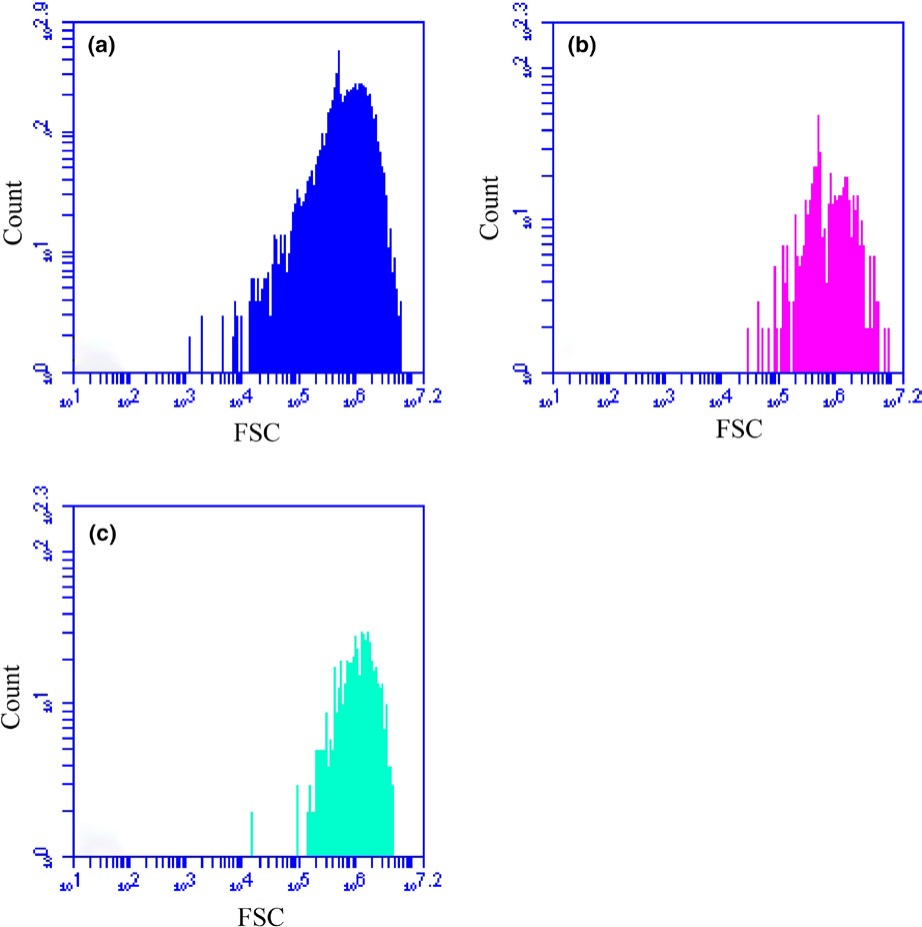
Forward scatter frequency (FSC) of (a) *Prochlorococcus*, (b) *Synechococcus,* and (c) picoeukaryotic phytoplankton in counting cells

Within the present study, three dominating populations, namely *Synechococcus*,* Prochlorococcus,* and picoeukaryotic phytoplankton, were manually distinguished by FCM (BD Accuri C6) according to their different amplitudes, shapes, and optical signals. The aforementioned references revealed that FSC was much more adaptable to establish the empirical relationship with cellular sizes of picophytoplankton (<2 μm). Thereafter, cellular diameters of them were enumerated by using the FCM based on their distinct FSC signatures. The histograms of example FSC frequency of three picophytoplankton groups are shown in Figure 2. We estimated the sizes of mean FSC frequency were of the order of the cell diameters of *Prochlorococcus*,* Synechococcus*, and picoeukaryotic phytoplankton, respectively. Otherwise, we presumably assumed that the optical signals of mean cell diameters of *Prochlorococcus* (0.6 μm) were similar to the normalized beads; subsequently, the cellular sizes with respect to biovolumes were estimated on the basis of the mean FSC frequency of *Prochlorococcus*,* Synechococcus*, and picoeukaryotic phytoplankton as measured relative to those of the mean cell diameters (Partensky, Hess, & Vaulot, 1999). The empirical relationship between the mean FSC (MFSC) and cell diameters (*d*_cell_) is shown as follows: *d*_cell_ = *d*_bead(*Prochlorococcus*)_(MFSC_cell_/MFSC_*Prochlorococcus*_). This hypothesis that was similar to the pattern of Blanchot et al. (2001) was an oversimplification, although the result needed to be considered cautiously and more proper calibration in further studies, the relationship was in accordance with those presently quoted.

Additionally, two different subclusters of *Synechococcus* were directly evidenced by two distinct peaks (bimodal distribution) in the histograms of the FSC frequency (Figure 2b), and possibly corresponding to the “*Synechococcus*I” and “*Synechococcus*II” cells (Zhao et al., 2013). Unfortunately, due to the absence of distinctive features and peak‐overlap, it was difficult to identify them by the FSC frequency. Therefore, these subclusters in the picophytoplanktonic fraction of the Indian Ocean were artificially combined and exclusively represented by *Synechococcus*.

## 3 RESULTS AND DISCUSSION

### 3.1 Cell size and biomass

**Figure 3:**
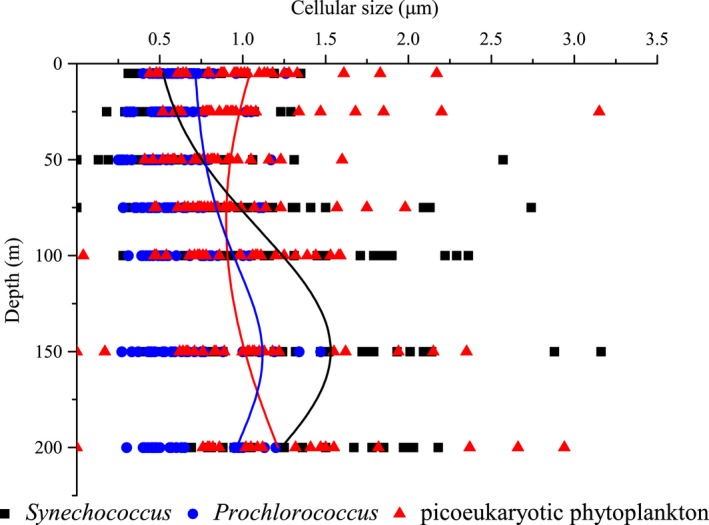
Vertical distributions of cell size in *Synechococcus*,* Prochlorococcus,* and picoeukaryotic phytoplankton

Results from the estimated mean FSC method indicated that mean cell diameter of *Prochlorococcus* (0.60 ± 0.22 μm) was the smallest, followed by *Synechococcus* (0.98 ± 0.44 μm) and picoeukaryotic phytoplankton (1.05 ± 0.30 μm). Our estimated cellular sizes for picophytoplankton were comparable with other estimates. For example, *Synechococcus* cell diameters well agreed with reported sizes of 0.74–1.22 μm, for water samples collected from the Sargasso Sea (DuRand et al., 2001). Using the same method who obtained a relationship between FSC and cell size on marine picophytoplankton cultures, Shalapyonok, Olson, and Shalapyonok (2001) estimated average values of 0.91–0.95 μm and 0.98–1.14 μm for *Synechococcus* in the surface mixed layer and below the mixed layer, respectively. However, our estimates of cellular size of picoeukaryotic phytoplankton were slight smaller than other estimates. Estimates of picoeukaryotic phytoplankton in equivalent spherical diameter based on other methods ranged from 1 to 2 μm, for example, 1.35–2.05 μm in the central Cantabrian Sea (Calvo‐Díaz, Morán, & Suárez, 2008) and 1.93–2.07 μm in equatorial Pacific (Blanchot et al., 2001). Overall, without accompanying independent measurements to use as a comparison, we are not able to judge whether our estimates are invalid. To clearly understand the vertical pattern, all the data points of picophytoplankton cellular size against depth were analyzed to plot related fitting curves (Figure 3). The three curves in Figure 3 represented vertical variations of cell size of three picophytoplankton groups, respectively. In particular, the observed vertical trend of cell size of picoeukaryotic phytoplankton was very similar to the pattern in the Arabian Sea (Shalapyonok et al., 2001), which showed that picoeukaryotic phytoplankton in the surface were slightly larger than those in the subsurface chlorophyll maximum. Diameter minimum for *Synechococcus* and *Prochlorococcus* occurred in upper waters, whereas larger cells were recorded near the 150‐m layer of water column (Figure 3). Generally, the variations in cellular diameter were even greater with depth in equatorial EIO during spring.

Picophytoplankton biomass could be measured directly from proximate analyses (such as chlorophyll, ATP, carbon, and nitrogen concentration) or indirectly from biovolume characteristics of the enumerated population (Frame & Hu, 1990; Hewes, Sakshaug, Reid, & olm‐Hansen, 1990; Hunter & Laws, 1981; Sun & Liu, 2003). Nevertheless, to separate *Prochlorococcus*,* Synechococcus,* and picoeukaryotic phytoplankton from other algae cells, and then to estimate the different biomass of them, respectively, using proximate analyses in situ were very difficult because of their considerably small cells. Determinations of proximate constituents such as levels of chlorophyll or detritus plus viable carbon were, therefore, inadequate measurements for use in studies on species or size‐class contributions to primary production (Sun, Liu, & Qian, 2000). For each picophytoplankton assemblage, the mean biovolume should be calculated from the mean value of these individual cell biovolumes, rather than directly from the mean cell diameter. In the equatorial EIO, the average abundances of *Prochlorococcus*,* Synechococcus* and picoeukaryotic phytoplankton were at the magnitude of 10^3^–10^5^ cells·ml^−1^ (Table 1); hence, it was a extremely complicated and heavy work to calculate the biovolume of each picophytoplanktonic cell. However, mean cell size differs greatly between species of picophytoplankton that suggests the mean cell size of species is very important with biomass (Agusti, Duarte, & Kalff, 1987; Liu, Chang, Tseng, Wen, & Liu, 2007; Marañón, 2015). Accordingly, Hillebrand, Dürselen, Kirschtel, Pollingher, and Zohary(1999) proposed that the biovolume can be calculated from the mean of measured cell diameters, not as a mean of a set of individually calculated biovolumes although there may be some errors. Indeed, the error sources within this study primarily came from the choice of mean cell diameter, in addition to the accuracy of measurement and consequent estimation of biovolume. When the two methods for mean biovolume calculation were compared, Sun and Liu (2003) found that although the latter method usually underestimated the variability, its trend had better agreement with increased measurements. Under most circumstances, the standard error was <5% of the mean biovolume after the measurement of 10 cells, we suggested that taking as many measurements as possible was better. As yet, the conversion of carbon content from biovolume based on mean cell diameter is the good way to estimate the gross biomass of picophytoplankton in the absence of direct unicellular size measurement. For example, Calvo‐Díaz and Morán (2006) obtained the mean diameters of the different groups of picophytoplankton by an empirical calibration, and thereafter assumed a spherical shape for all groups to estimate biomass by using the method of biovolume‐to‐carbon conversion in the southern Bay of Biscay; Chiang, Kuo, Chang, Wang, and Gong(2002) computed the mean cell volume based on the approximately coccoid shape of *Synechococcus* cells to estimate biomass in the East China Sea; Blanchot et al.(2001) attempted a very practical way for the estimation of mean cell size to calculate cellular carbon in the equatorial Pacific. Thus, the mean measured cell diameter of picophytoplankton could be used to calculate biovolume in routine analysis. The carbon biomass estimates of *Synechococcus*,* Prochlorococcus*, and picoeukaryotic phytoplankton for this study area were roughly calculated by using the mean carbon content per cell multiplied by cell abundance for each of these groups. Preferentially, we assigned the geometric shape for a picophytoplanktonic cell as a sphere (Chisholm et al., 1992). The cell diameter (*d*) was converted to biovolume (*V*) using a predictive equation (Sun et al., 2000): $\begin{array}{c} V=\frac{4}{3}\pi {\left(\frac{d}{2}\right)}^{3} \end{array}$. Then, an average biovolume per cell was converted to the average carbon value per cell using the empirical relationship (DuRand et al., 2001; Eppley, Reid, & Strickland, 1970): log*C* = 0.94 × log*V* − 0.60. Picophytoplankton abundance (Table 1) was converted to biomass using the resulting volume to carbon conversion factors: 32 fg·C·cell^−1^ for *Prochlorococcus*, 129 fg·C·cell^−1^ for *Synechococcus*, and a carbon content of 160 fg·C·cell^−1^ for picoeukaryotic phytoplankton. Finally, the mean carbon concentrations reached 0.64 μg·C·L^−1^ for *Prochlorococcus*, 0.34 μg·C·L^−1^ for *Synechococcus*, and 0.20 μg·C·L^−1^ for picoeukaryotic phytoplankton in this study.

**Table 1:** Mean values of picophytoplankton abundance (cells·ml^−1^) in whole equatorial EIO and four main transects (T)

Study area/factors	*Synechococcus* (×10^3^)	*Prochlorococcus* (×10^4^)	Picoeukaryotic phytoplankton (×10^3^)
Whole area	2.65 ± 1.54	2.02 ± 1.07	1.26 ± 1.07
T‐A	2.57 ± 0.58	1.74 ± 1.15	1.65 ± 1.47
T‐B	1.18 ± 0.62	1.53 ± 0.79	0.71 ± 0.42
T‐C	1.12 ± 0.24	1.21 ± 0.49	0.66 ± 0.29
T‐D	1.66 ± 1.05	2.24 ± 1.28	1.24 ± 0.85

**Figure 4:**
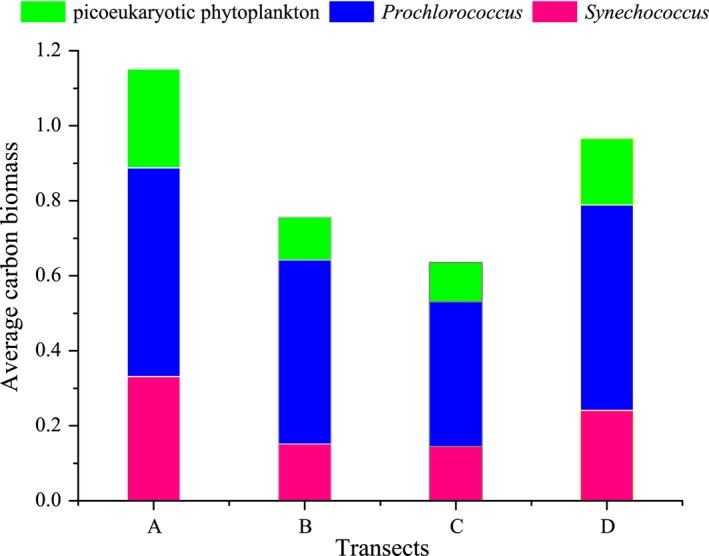
The average carbon biomass (μg·C·L^−1^) of *Synechococcus*,* Prochlorococcus,* and picoeukaryotic phytoplankton along transects A, B, C, and D

**Figure 5:**
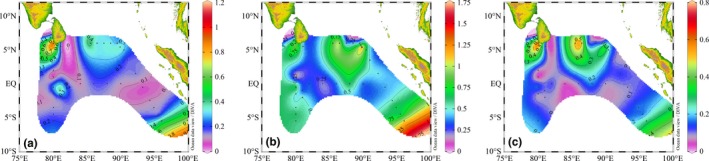
Horizontal distribution of the average carbon biomass (μg·C·L^−1^) for (a) *Synechococcus*, (b) *Prochlorococcus,* and (c) picoeukaryotic phytoplankton

The estimated average carbon biomass of *Synechococcus*,* Prochlorococcus,* and picoeukaryotic phytoplankton tended to have different distribution patterns (Figures 4 and 5). Owing to their high abundance, the relative contribution of *Prochlorococcus* to the total picophytoplankton carbon biomass was higher than the other two groups, indicating that *Prochlorococcus* was the dominant component in terms of average carbon biomass throughout the equatorial EIO. Moreover, the average carbon biomass of three picophytoplankton groups were primarily concentrated in section A, where was profoundly influenced by surface freshwater from the Bay of Bengal runoffs (Sengupta, Bharath Raj, & Shenoi, 2006). Although *Synechococcus* and picoeukaryotic phytoplankton had larger cellular sizes, their abundance was approximately 1–2 orders of magnitude less abundant than *Prochlorococcus*. Overall, this result agreed with those reported in the regions of tropical and subtropical Pacific Ocean, *Prochlorococcus* was the most abundant photosynthetic organism, even accounting for 65% of the total picoplankton biomass, whereas *Synechococcus* and picoeukaryotic phytoplankton constituted less than 35% of the biomass (Blanchot & Rodier, 1996; Campbell, Nolla, & Vaulot, 1994; Charpy & Blanchot, 1998). Interestingly, some previous studies reported that *Synechococcus* could fix an order of magnitude more carbon than *Prochlorococcus* cells due to *Synechococcus* was slightly larger than *Prochlorococcus*, indicating that the relative biomass contributions of three picophytoplankton groups were not simply determined by abundance but also changes in cellular size (Flombaum et al., 2013). However, our finding demonstrated that the distinct biomass contributions of picophytoplankton in the equatorial EIO appeared to be affected by abundance, but not changes in cellular size. In addition to the southern Bay of Bengal, the maximal carbon biomass of *Synechococcus* and picoeukaryotic phytoplankton co‐occurred around the coastal zones of Sri Lanka island and Sumatra in horizontal distribution, where were potentially contributed by the freshwater discharging from coastal currents and coastal upwelling, respectively. Similarly, slightly high carbon biomass of *Prochlorococcus* were distributed around the coastal zones of Sri Lanka and the southern Bay of Bengal, but they, in particular, presented the highest carbon biomass in the coastal upwelling zones of Sumatra.

### 3.2 Factors controlling on cell size and biomass

**Figure 6:**
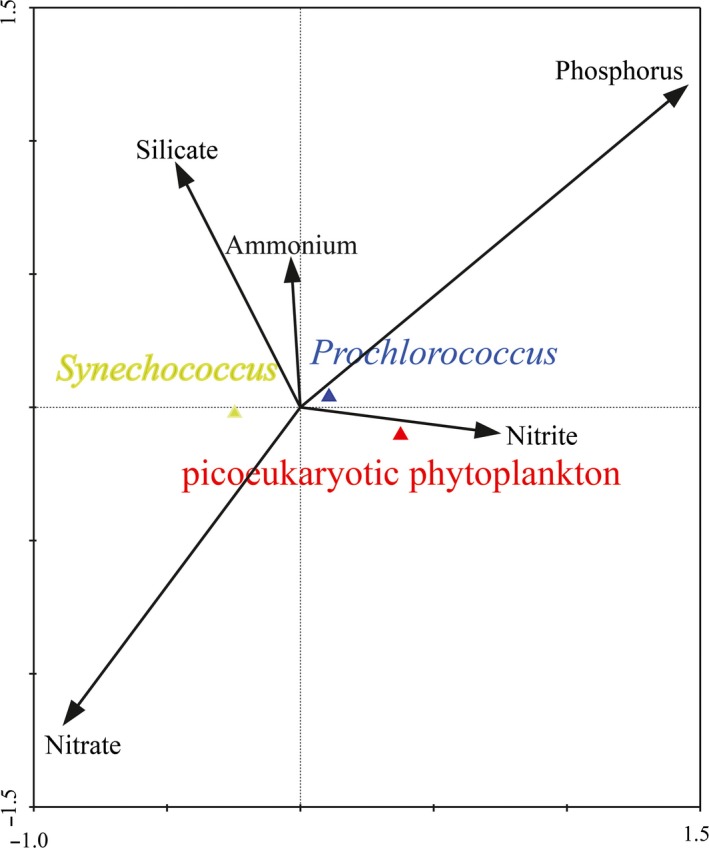
Canonical correspondence analysis of nutrient variables with the carbon biomass for three picophytoplankton groups

As reported previously for picophytoplankton, the differences in chlorophyll fluorescence signals (cellular sizes) observed with depth in water column were most likely due to photoacclimation (i.e., reduction of the pigment content at high light levels), cellular division, changes in quantum yield, and shifts in species composition (Campbell & Vaulot, 1993). Specifically, marine environmental variables such as temperature, light, and nutrient availability usually had apparent effects on the cellular sizes of picophytoplankton (Chen et al., 2011). Taken together, the cellular size of picophytoplankton appears to be under complex physiological and environmental control. In equatorial EIO, there were less nutrients in upper waters (5–50 m) during spring monsoon (Table 2). Figure 6 shows the Canonical Correspondence Analysis (CCA) of average carbon biomass of *Prochlorococcus*,* Synechococcus,* and picoeukaryotic phytoplankton with nutrient variables. Combined with Table 3, no significant correlations were found between *Synechococcus* biomass and DIP, ammonium, and nitrite (*p *>* *.05). However, *Synechococcus* biomass were positively correlated with nitrate and DSI (*p *<* *.01), suggesting that *Synechococcus* biomass including cellular sizes was favored by the optimal nitrate and DSI concentrations. Recently, Baines et al.(2012) discovered natural populations of marine cyanobacteria of the genus *Synechococcus* contained a significant amount of the element silicon. Demonstration of an obligate need for Si in *Synechococcus* would add a new dimension to their nutrient physiology and to the suite of resources influencing *Synechococcus* abundance in nature. Thus, the DSI might also affect *Synechococcus* biomass to carbon cycling as our CCA shown that could be facilitated by silicon ballasting of aggregates and fecal pellets containing *Synechococcus* if cells contained polymerized silica as recently reported (Brzezinski et al., 2017). In contrast with *Synechococcus*,* Prochlorococcus,* and picoeukaryotic phytoplankton biomass showed strong negative correlations with nitrate and DSI, whereas they were closely related with DIP and nitrite (*p *<* *.01), indicating that they were mostly profited from the environment in condition with high DIP and nitrite concentrations. The DIP concentration was so low in the equatorial EIO that might be a limiting factor for the growth of *Prochlorococcus* and picoeukaryotic phytoplankton (Table 2). Moore et al. (2002) similarly demonstrated that *Prochlorococcus* biomass was closely related to the nitrite concentration owing to the high‐B/A ecotypes had homologs of genes required for nitrite utilization; hence, nitrite could be available N source for such subpopulations. Conclusively, different positive correlations with each environmental variable indicated that nutrients were the crucial factors in regulating their biomass and cellular sizes. The cells of picophytoplankton in upper waters are growing slower when the nutrient levels are lower, whereas cells are on average smaller when they grow slower (DuRand et al., 2001). Results from the size fractionation method revealed that the sizes of *Prochlorococcus* and *Synechococcus* in the deep euphotic layers were significantly larger than those in the upper euphotic layers (Liu et al., 2007), and these differences in cell size were attributed to the growth rate at different nutrient concentrations. In dilution experiments, Liu et al. (1998) reported that the growth rate of *Synechococcus* was much higher than *Prochlorococcus* with nutrient availability. Furthermore, Shalapyonok et al. (2001) found that the cyanobacteria cells inhabiting the top layer were smaller than those inhabiting the deeper layer, which might be related to nutrient depletion, as well as photoacclimation. Actually, this hypothesis that lower nutrient concentration in upper waters induced smaller size was typically suitable for picophytoplankton in equatorial EIO (Figure 3). Although their cells were remarkably small in the upper waters, the smaller cells of *Prochlorococcus* and *Synechococcus* had advantages relative to larger picoeukaryotic phytoplankton cells in terms of resource acquisition and utilization in growth and reproduction, because of their very large surface area per unit volume and minimal diffusion boundary layer thickness (Raven, 1998). Collectively, the variations of cellular sizes in upper waters were closely related to the nutrient availability.

**Table 2:** Average nutrient concentrations (μmol·L^−1^) of different water layers over 0–200 m during spring 2015. BDL: below detection limits

Depth/Parameter		Ammonium	Phosphate	Nitrate	Nitrite	Silicate
5 m	Range	0.14–2.69	BDL‐0.35	BDL‐14.50	BDL‐1.31	0.29–1.47
	Mean	0.63 ± 0.54	0.11 ± 0.07	0.99 ± 2.31	0.18 ± 0.29	0.86 ± 0.31
50 m	Range	0.01–0.71	0.06–0.85	BDL‐15.38	BDL–1.09	0.07–3.47
	Mean	0.27 ± 0.16	0.23 ± 0.17	1.73 ± 3.02	0.15 ± 0.25	0.98 ± 0.64
100 m	Range	0.06–1.46	0.46–2.66	4.55–33.43	0.03–0.31	0.96–15.37
	Mean	0.55 ± 0.28	1.23 ± 0.46	14.35 ± 6.62	0.16 ± 0.07	4.04 ± 2.66
150 m	Range	0.07–0.98	0.42–2.45	4.08–49.75	BDL‐0.14	2.20–17.68
	Mean	0.45 ± 0.21	1.12 ± 0.61	16.27 ± 10.93	0.06 ± 0.05	7.16 ± 3.76
200 m	Range	0.05–1.24	0.45–2.92	5.80–44.00	BDL‐0.29	3.35–54.69
	Mean	0.43 ± 0.25	1.20 ± 0.74	19.49 ± 10.27	0.03 ± 0.06	11.24 ± 11.63

**Table 3:** Spearman's rank correlation coefficients between environmental factors and carbon biomass of three picophytoplankton groups

	DIP	Ammonium	Nitrite	DSI	Nitrate
*Prochlorococcus*	0.139^b^	−0.109	0.301^b^	−0.344^b^	−0.321^b^
*Synechococcus*	−0.597	0.059	−0.159	0.542^b^	0.580^b^
Picoeukaryotic phytoplankton	−0.177	−0.069	0.502^b^	−0.248^a^	−0.184

a Correlation is significant at the .05 level (two‐tailed).

b Correlation is significant at the .01 level (two‐tailed).

**Figure 7:**
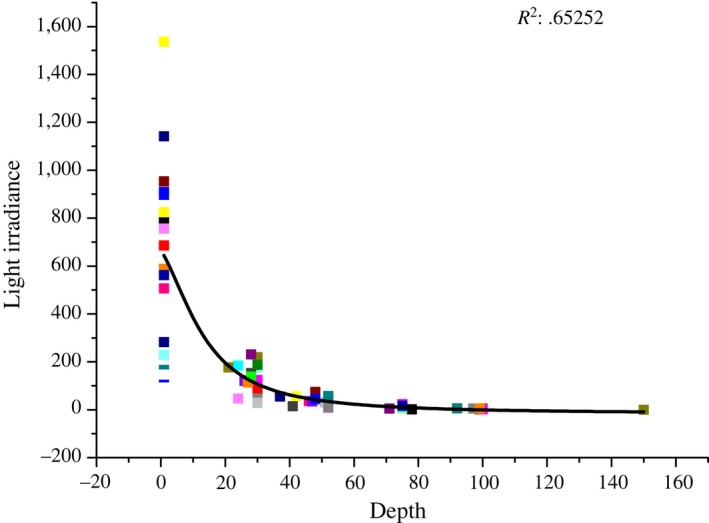
Nonlinear fitting curve between depth (m) and light irradiance (μmol photons m^−2^·s^−1^)

**Figure 8:**
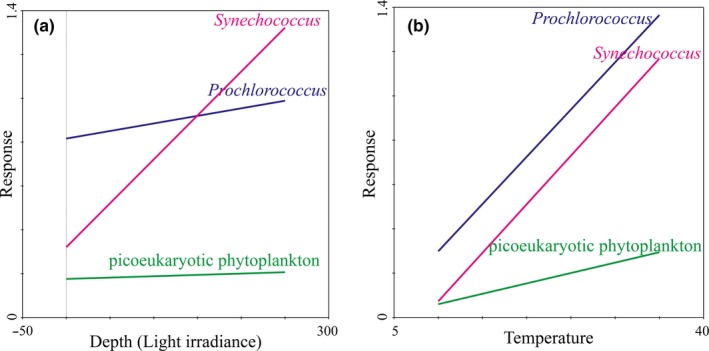
Different response relations between cellular size and light levels (depth (m)) and temperature (°C) for three picophytoplankton groups

Due to phytoplankton that grow deeper in the water column need more pigment per cell to compensate for the decreased light levels, their cellular sizes are broadly larger under lower light intensity (Goericke & Welschmeyer, 1998). Den Engh et al. (2017) also presented that chlorophyll fluorescence, and to a lesser extent forward light scatter (an approximate proxy for cell size), increased with depth. The above‐reported increase in the relative cellular sizes of phytoplankton with decreasing light levels might be fully applicable to picophytoplankton in our dataset. Vertically, *Prochlorococcus*,* Synechococcus*, and picoeukaryotic phytoplankton inhabiting in the deeper layers were larger and had higher chlorophyll fluorescence than those inhabiting in the upper layers (Figure 3). It is well known that the PAR values vary greatly at different times of 1 day, but the abundance and cell size of picophytoplankton will not vary too much although they are potentially influenced by the light availability. Furthermore, the refraction of light is largely dependent on the particles, suspended sediments, and also color dissolved organic matter. Accordingly, it was impossible to establish the linear correlation between cell size and light level directly owing to the variability of light irradiance. According to the nonlinear fitting curve between depth and light irradiance (Figure 7), the light intensity decreased with the increasing depth in the equatorial EIO (*R* ^2^ = .65252), indicating that the decrease in light irradiance could be effectively represented by the increasing depth. Consequently, we established the linear response between cell size and the increasing depth (representing the decreasing light irradiance) to show different response relations of cell size and light intensity. Figure 8 shows the distinct response relations between cellular size and depth (light levels) and temperature for three picophytoplankton groups in the equatorial EIO. According to our analysis of response, the slopes of these lines for cell size of *Prochlorococcus* and *Synechococcus* to depth and temperature were relatively high, which represented high response levels of cell size to light levels and temperature. Thus, this observation indicated the vertical patterns of *Prochlorococcus* and *Synechococcus* in cellular sizes were the results of combined effects of photoacclimation and temperature (Figure 8). Similarly, Goericke and Welschmeyer (1998) proposed that the increase in cellular size with depth might be attributed to photoacclimation because of the need to synthesize more proteins and pigments to capture limiting photons. Thus, the organisms adjusted their chlorophyll content, size, and shape to compensate for the changes in irradiance with increasing depth. In laboratory batch cultures, Burbage and Binder (2007) reported that the cellular sizes of oceanic *Prochlorococcus* MIT9312 and *Synechococcus* WH8103 grown under high light were smaller than the sizes of cells grown under low light in semi‐continuous cultures. Otherwise, Montagnes and Franklin (2001) suggested that lower temperature could lead to larger size for *Prochlorococcus* and *Synechococcus* because of the growth dilution, with roughly 4% increase of cellular volume per centigrade decrease. Overall, the cellular sizes of *Synechococcus* and *Prochlorococcus* were larger under deep layer in the equatorial EIO, which was primarily attributed to the different responses to lower temperature and reduced light intensity. For *Prochlorococcus*, low‐B/A isolates are able to grow maximally at high light intensity, while high‐B/A isolates acclimate to the low light condition of the deep euphotic zone (Moore & Chisholm, 1999). Changes in the dominance of different ecotypes were consequently critical in explaining the vertical variations of their cellular sizes in addition to temperature and light availability in deeper waters. For picoeukaryotic phytoplankton, however, due to the lower capacity to photoacclimate controlled by physiological differences, their cellular sizes were larger occurring in low light layers (Campbell et al., 1997). Similarly, the pronounced increase with depth in picoeukaryotic phytoplankton size between the depth of 100 m and 200 m was the result of a dramatic change in light intensity.

Spatially, most of coastal waters around the Sri Lanka island are influenced by the increases of pollution and eutrophication. Simultaneously, the East India Coastal Current (EICC) along the western boundary of the Bay of Bengal flows equatorward and bifurcates east of the Sri Lanka island, but one bifurcation of its source waters characterized by nutrient enrichment continues along the coast of Sri Lanka island (Vinayachandran et al., 2005). Therefore, this studied coastal area is abundant in nutrients and suitable for picophytoplankton development. The CCA analysis revealed that significant correlations were found between the average carbon biomass of *Prochlorococcus*,* Synechococcus,* and picoeukaryotic phytoplankton and nutrient variables, reinforcing the notion that nutrient supply was the key limiting factor for the spatial distribution of average carbon biomass. Therefore, such contaminants loading from coastal currents and freshwater discharging from the EICC with high nutrients contributed to the fairly high average carbon biomass of three picophytoplankton groups in coastal waters of the Sri Lanka island. Surface temperature surrounding the Sumatra was below 29°C with a comparatively high salinity of approximately 34 during spring 2015, indicating that its hydrographic properties were prominently attributed to the coastal upwelling. The co‐occurrence of high average carbon biomass for picophytoplankton in coastal upwelling zones nearby the Sumatra was not surprisingly owing to the high nutrient availability. Waters of very low salinity and temperature (32°C and 29°C, respectively) were presented in the northern Bay of Bengal, thereby indicating that they were dramatically influenced by freshwater from the Bay of Bengal runoffs. As such, slightly high average carbon biomass of three picophytoplankton groups co‐occurred in the southern Bay of Bengal due to they could benefit from the increased nutrient availability carried by the Bay of Bengal runoffs (Mukhopadhyay, Biswas, De, & Jana, 2006). Collectively, as a result of differential responses of picophytoplankton in average carbon biomass to the changes in physical and chemical environments induced by the variable circulations and water masses, we observed very different spatial patterns of average carbon biomass for these three picophytoplankton groups.

## CONFLICT OF INTEREST

The authors declare that the research was conducted in the absence of any commercial or financial relationships that could be construed as a potential conflict of interest.
